# Raptor hunted by caspases

**DOI:** 10.1038/cddis.2016.153

**Published:** 2016-06-02

**Authors:** R Martin, M Thome, F Martinon, N Fasel

**Affiliations:** 1 Department of Biochemistry, University of Lausanne, Chemin des Boveresses 155, Epalinges, Vaud 1066, Switzerland

Emergence of survival strategies is a key step for organisms during evolution. The capacity to adapt from nutrient-rich to nutrient-poor environments led to the appearance of protein complexes regulating anabolic and catabolic pathways. The evolutionarily conserved kinase mTOR (mechanistic target of rapamycin) emerged as a crucial and central protein for regulating many different cellular functions in response to environmental changes.

The kinase mTOR interacts with different partners to form two distinct complexes, which differentiate from one to the other by the presence of the regulatory-associated protein of mTOR (raptor)^1^ in the mTORC1 and the rapamycin-insensitive companion of mTOR (rictor)^2^ in the mTORC2. These two mTOR complexes integrate intracellular and extracellular signals, such as presence of growth factors or amino acids, energy status, oxygen and stress, which lead to modulation of mTORC1/2-dependent signaling events. The mTORC1 regulates key cellular pathways like protein synthesis (through phosphorylation of S6K and 4E-BP1), autophagy, lipid synthesis, energy metabolism and lysosome formation, whereas mTORC2 controls the organization of the cytoskeleton, cell survival, apoptosis and metabolism.^3^ Acting together in a nutrient-rich environment, these two mTOR complexes positively regulate cell anabolism and, at the same time, negatively regulate cell catabolism.

More than 20 years after the discovery of the mTOR complexes, novel aspects of mTOR regulation are still emerging and contribute to the growing complexity of these metabolic sensors. The activation level of mTORC1 is tightly controlled by many different protein signaling cascades, leading to adaptation to environmental changes. Precise regulation of mTORC1 activity is crucial, since its hyperactivation has been linked to many human disorders, including metabolic diseases (type 2 diabetes, insulin resistance), neurological diseases and cancers.^3, 4^

mTORC1 hyperactivation in cancers has been proposed to contribute to uncontrolled cell proliferation.^5^ This led to the development of mTORC1 targeting inhibitors, such as rapamycin analogs (rapalogs), aimed at blocking proliferation of cancer cells. While common anticancer drugs also inhibit mTORC1,^6, 7^ the mechanisms involved are not fully understood.

In our recent *Cell Death Discovery* publication (doi:10.1038/cddiscovery.2016.24), we show that treatment with different chemotherapeutic drugs triggers proteolytic processing of the mTORC1-specific scaffolding protein raptor. Anti-cancer compounds targeting mTORC1, such as rapamycin, curcumin, etoposide, cisplatin, staurosporine and FasL, initiated apoptosis in various lymphoma cell lines and promoted the detection of a raptor-cleaved fragment of 100 kDa. This proteolytic event was blunted in the presence of specific caspase inhibitors, indicating that caspases were involved in this process. *In vitro* experiments demonstrated that active recombinant caspase-6 cleaved a recombinant raptor protein as well as the endogenous raptor in Jurkat T-cell lysates. Moreover, raptor cleavage was decreased in caspase-6 knockout (KO) cells, further indicating the important role of caspase-6 in raptor processing upon treatment with various drugs. However, at this point, we cannot exclude that other apoptotic caspases could play a redundant role in the cleavage of raptor due to the incomplete inhibition of raptor proteolytic cleavage in caspase-6 KO cells.

The caspase-specific cleavable residues of raptor were investigated by mutagenesis and mass spectrometry. At least two cleavage sites were identified, one within the N-terminal region and one in the C-terminal part, both yielding the predicted 100-kDa cleavage fragment of raptor after treatment of cancer cells with chemotherapeutic drugs. Interestingly, raptor cleavage induced a decrease in the interaction between raptor and the mTOR kinase, suggesting that raptor processing could affect the signalosome of the mTORC1 (Figure 1). In addition, we observed a striking decrease of mTORC1 kinase activity towards S6K and 4E-BP1, which correlated with the processing of raptor upon cell death induction. Furthermore, use of the raptor cleavage-resistant mutant indicated that proteolytic processing of raptor participated in the cell death sensitivity, which is essential for impairing mTORC1 functions and promoting cell death signaling.

Recent structural evidences support a model in which the mTORC1 forms a dimeric circle-like structure, composed of two raptor molecules and two mTOR molecules.^8, 9^ The close interactions between raptor and the mTOR kinase are essential for the stability of the mTORC1, but also for the recruitment of mTOR substrates towards the mTOR kinase domain by the action of raptor.

Raptor being the cement of the mTORC1, the identification of its cleavage by caspases reveals an important new aspect of mTORC1 regulation. Several reports have highlighted the dissociation of raptor from mTOR after incubation with rapamycin^10^ or curcumin,^11^ two drugs affecting the proliferation of cancer cells. Our findings suggest that the apoptotic cascade, triggered by different drugs, efficiently inhibits mTORC1 by cleaving raptor, which perturbates the recruitment of mTOR substrates by raptor. Moreover, similarly to the translation initiation factor, 4E-BP1, which can be cleaved by caspases during apoptosis,^12^ the generated cleaved fragment of raptor could act as a proapoptotic fragment, competing with the remaining uncleaved and mTOR-bound raptor for the mTOR substrates and thus inhibiting mTORC1 signalosome. Collectively, these findings reveal an essential role of caspases in repressing mTORC1 functions during apoptosis, which irreversibly leads to cell death.

Our findings not only highlight a mechanism of mTOR regulation but also identify a new target for caspase-6 during apoptosis. Since this atypical caspase has been implicated in neurological diseases, such as Alzheimer's, Parkinson's and Huntington's diseases, caspase-6-mediated mTORC1 regulation may be relevant in these age-related diseases.^13^ Active caspase-6 has been correlated with Alzheimer's disease progression, due to its role in the cleavage of cytoskeletal proteins like *α*-tubulin, *α*-actinin-4 and spinophilin, which impairs the cytoskeleton network of neurons. Furthermore, caspase-6 can also cleave Tau, huntingtin and the amyloid precursor protein, also leading to axonal degeneration.^14, 15^ The discovery of the caspase-6-mediated proteolytic processing of raptor highlights another level of regulation of the mTORC1, which could be important for neurotoxicity and neurodegeneration.

In conclusion, we propose a new caspase-6- and raptor-dependent mechanism of mTORC1 inhibition, which could be exploited to develop new chemotherapeutic strategies. In this context, a synergistic effect could be obtained by combining mTOR kinase inhibitors with anti-cancer drug-activating caspases, giving rise to a strong repression of the mTORC1 and thus offering new therapeutic perspectives for a large range of human diseases.

## Figures and Tables

**Figure 1:**
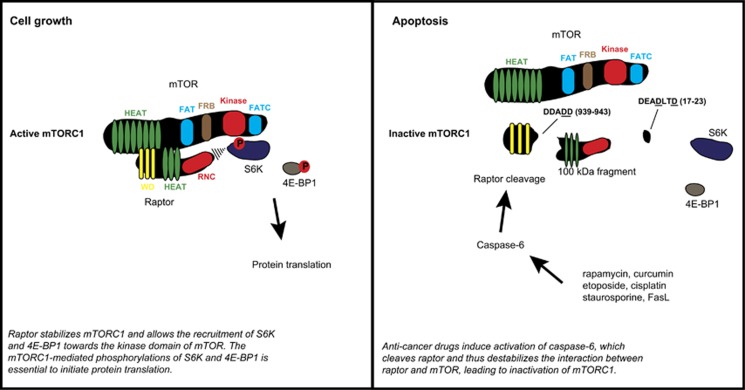
Mechanisms of the caspase-6-driven inhibition of mTORC1. The left panel shows the stabilizing interaction between raptor and mTOR and its crucial role in regulating mTOR kinase activity towards S6K and 4E-BP1, both of which participate in the regulation of protein translation during cell growth. The right panel represents molecular mechanisms triggered by chemotherapeutic drugs. Rapamycin, curcumin, etoposide, cisplatin, staurosporine and FasL induce activation of caspase-6, among other caspases, which cleaves the mTORC1-specific protein, raptor, at two precise residues, thus weakening its interaction with mTOR. The caspase-6-mediated proteolysis of raptor perturbates the mTOR kinase activity and participates in the apoptotic cascade. FAT, focal adhesion targeting; FATC, C-terminal focal adhesion targeting; FRB, FKBP12-rapamycin binding; HEAT, huntingtin, elongation factor 3 (EF3), protein phosphatase 2A (PP2A), and the yeast kinase TOR1; RNC, raptor N-terminal conserved domain; WD, tryptophan-aspartic acid (WD) dipeptide
